# Predialysis education in practice: a questionnaire survey of centres with established programmes

**DOI:** 10.1186/1756-0500-7-730

**Published:** 2014-10-17

**Authors:** Mario Prieto-Velasco, Corinne Isnard Bagnis, Jessica Dean, Tony Goovaerts, Stefan Melander, Andrew Mooney, Eva-Lena Nilsson, Peter Rutherford, Carmen Trujillo, Roberto Zambon, Carlo Crepaldi

**Affiliations:** Unidad de Nefrología, Complejo Asistencial Universitario de León, León, Spain; Service de Néphrology, Groupe Hospitalier Pitié-Salpêtrière et Chaire de Recherche en Education Thérapeutique, Université Pierre et Marie Curie, Paris, France; Department of Clinical Health Psychology, Salford Royal Hospital, Salford, M6 8HD UK; Cliniques Universitaires St. Luc, Service de Néphrologie, Brussels, Belgium; Department of Nephrology, University Hospital of Linköping, Linköping, Sweden; Renal Unit, St James’s University Hospital, Leeds Teaching Hospitals NHS Trust, Leeds, LS9 7TF UK; Department of Nephrology and Transplantation, Skånes University Hospital, Malmö, Sweden; Baxter-Gambro Renal, Zurich, Switzerland; Unidad clínica de Gestión de Nefrología, Hospital Regional Carlos Haya, Malaga, Spain; Unità Operativa di Nefrologia, Dialisi e Trapianto, Ospedale San Bortolo, ULSS106 Vicenza, Italy

**Keywords:** Predialysis, Renal replacement therapy, Education, Chronic kidney disease, Questionnaire

## Abstract

**Background:**

There is growing evidence that renal replacement therapy option education (RRTOE) can result in enhanced quality of life, improved clinical outcomes, and reduced health care costs. However, there is still no detailed guidance on the optimal way to run such programmes. To help address this knowledge gap, an expert meeting was held in March 2013 to formulate a position statement on optimal ways to run RRTOE. Experts were selected from units that had extensive experience in RRTOE or were performing research in this field. Before the meeting, experts completed a pilot questionnaire on RRTOE in their own units. They also prepared feedback on how to modify this questionnaire for a large-scale study.

**Methods:**

A pilot, web-based questionnaire was used to obtain information on: the renal unit and patients, the education team, RRTOE processes and content, how quality is assessed, and funding.

**Results:**

Four nurses, 5 nephrologists and 1 clinical psychologist (9 renal units; 6 EU countries) participated. Nurses were almost always responsible for organising RRTOE. Nephrologists spent 7.5% (median) of their time on RRTOE. Education for the patient and family began several months before dialysis or according to disease progression. Key topics such as the ‘impact of the disease’ were covered by every unit, but only a few units described all dialysis modalities. Visits to the unit were almost always arranged. Materials came in a wide variety of forms and from a wide range of sources. Group education sessions were used in 3/9 centres. Expectations on the timing of patients’ decisions on modality and permanent access differed substantially between centres. Common quality assurance measures were: patient satisfaction, course attendance, updated materials. Only 1 unit had a dedicated budget.

**Conclusions:**

There were substantial variations in how RRTOE is run between the units. A modified version of this questionnaire will be used to assess RRTOE at a European level.

**Electronic supplementary material:**

The online version of this article (doi:10.1186/1756-0500-7-730) contains supplementary material, which is available to authorized users.

## Background

In Europe, chronic kidney disease (CKD) has a similar prevalence to diabetes [1]. Once CKD has developed into end-stage renal disease (ESRD), most patients will be treated using transplantation or dialysis, and some patients will be managed conservatively. In around 80% of dialysis patients, there are no medical grounds to indicate whether haemodialysis (HD) or peritoneal dialysis (PD) would be preferable [2]. Thus, current guidelines recommend that renal replacement therapy (RRT) units should provide access to all RRT modalities, along with well-balanced information on the modalities presented in a structured programme [3]. This would allow the patient to choose the option best suited to their individual needs. In practice however, large numbers of ESRD patients fail to receive such an educational programme [4, 5].

The benefits of RRT option education (RRTOE) can be quantified in medical and financial outcomes. In two Canadian studies, RRTOE was shown to reduce urgent dialysis starts, reduce time spent in hospital, and improve resource utilisation [6]. Cost savings were estimated to be over $4,000 (Canadian) per patient in 1993. Other studies have shown RRTOE to result in earlier placement of permanent vascular access [7], a greater likelihood of choosing a self-care modality [8], extended time to requiring dialysis [9] and reduced mortality [10].

Given the benefits of RRTOE, such programmes will become more common, and mandatory guidelines have been introduced in some countries. However, current guidelines: (1) lack the necessary detail required to design and run a programme [3, 11–14]; (2) often focus primarily on one particular aspect of a programme (e.g. enrolment criteria [14]); (3) are not always specific to CKD [15].

The sparse data available on how these guidelines are implemented in practice indicate that there is little standardisation or consensus on designing and running an RRTOE programme (Van den Bosch et al., Review of Predialysis Education Programmes: a Need for Standardization. Submitted). For example, in the UK alone in 2007, at least 31 different leaflets on dialysis were used in such programmes [16], and the majority had extremely poor readability.

The main knowledge gaps in the field of RRTOE are: (1) How is RRTOE being run? (2) How should RRTOE be run? To consider these questions, an expert meeting was held in Zurich, March 2013. Experts were selected from units that had extensive experience in RRTOE or were performing research in this field.

To help address the first knowledge gap, the experts completed a pilot questionnaire on their own RRTOE programmes, and provided detailed feedback on the questionnaire to make it suitable for a large-scale European survey (scheduled for 2014). To address the second knowledge gap, the experts offered practical advice on all aspects of RRTOE, from both the literature and their experience, to formulate a consensus statement. This has recently been published [17], along with a companion paper for nurses [18].

The current paper presents the two key outcomes of the expert meeting concerning the first knowledge gap: (1) Questionnaire results on how RROTE is run in the experts’ units. We believe these results will be an interesting reference point for health care professionals (HCPs) running RRTOE programmes ("Results" section). (2) Recommended improvements to the questionnaire for large-scale roll-out. We believe that this will be of interest to both researchers in the area and those interested in the results from the upcoming European survey ("Feedback on the questionnaire" section).

## Methods

### Participants

Six nephrologists, 8 nurses, and 1 clinical psychologist from 12 renal units were contacted, and invited to participate in the expert meeting. Selected units had either extensive experience with RRTOE or included at least one member of staff performing research on RRTOE. Upon accepting the invitation, experts were sent a link to the pilot questionnaire and guidelines on the feedback required.

### Design and administration of the pilot questionnaire

The pilot questionnaire was principally constructed by a single author (P.R.). The purpose was to gather detailed information on all aspects of RRTOE. It was intended that this questionnaire would undergo refinement following the experts’ feedback.

The questionnaire was split into 2 main sections. The first section was designed to obtain information on the renal unit, staff and patients in 2012. The second section was designed to obtain information on the RRTOE programme itself. The complete questionnaire can be found in an Additional file 1, and a breakdown of the sub-sections is presented below:

-  **Section 1: Renal unit, its services and patient population**
  - ○ RRT modalities offered at the unit
  - ○ Patient population
  - ○ Uptakes of RRT modalities and RRTOE
  - ○ Staff in RRT unit

-  **Section 2: Description of the RRTOE programme**

- ○ Staff involved in RRTOE
- ○ Starting RRTOE and enrolment criteria
- ○ Content and structure of RRTOE
  - ▪ Topics covered
  - ▪ Visits and meetings
  - ▪ Formal decision-making process
  - ▪ Materials
  - ▪ Setting
- ○ Funding
- ○ Quality assurance
- ○ Factors perceived to be important

Experts at the participating renal units filled out the questionnaire online using a web-based survey (http://www.surveymonkey.com).

As this questionnaire was designed solely to gather information on the RRTOE programmes and no identifiable patient data were collected, ethics committee approval was not required.

### Statistics

Results were analysed using descriptive statistics (percentage; median and range).

## Results

Unless otherwise indicated, answers to each question were provided by all participating units.

### Participating units

Four nurses, 5 nephrologists and 1 clinical psychologist from 9 renal units agreed to participate. Two units each were located in the UK, Spain and Sweden. The remaining 3 units were located in France, Belgium and Italy.

### Section 1: Renal unit, its services and patient population

#### RRT modalities offered at the unit

All units offer in-centre HD, automated peritoneal dialysis (APD), and continuous ambulatory peritoneal dialysis (CAPD). 6 units offer transplantation; 7 offer assisted PD; 5 offer home HD; 4 offer self-care HD. Full details are given in Table 1.

**Table 1:** **Details of each unit’s patient population, RRT modalities and staff**

	Transplant patients (n)	Dialysis patients (n)		Dialysis patients receiving different RRT modalities (%)						Staff (full time equivalents)					
	Prevalent (2012)	Prevalent (2012)	Incident (2012)	In-centre HD	Self-care HD	HHD	APD	CAPD	Asst. PD	Nephrologists	Nurses	Dieticians	Psychologists	Social workers	Other
**Centre 1**	807	639	120	80%	3%	2%	11%	2%	1%	9	240	3	2	1	2^a^
**Centre 2**	1,500	90	118	53%	n.o.	n.o.	27%	11%	9%	12	30	1	0.4	1	0
**Centre 3**	200 ^n.o.^	200	45	80%	n.o.	n.o.	10%	10%	n.o.	2	25	0.5	0.5	0.4	0
**Centre 4**	330	290	75	59%	n.o.	n.o.	10%	24%	7%	12	50	0	0	0	4^b^
**Centre 5**	150 ^n.o.^	116	25	60%	12%	12%	3%	11%	1%	7.5	40	1	0	1	0
**Centre 6**	1,350	793	123	95%	n.o.	0%	4%	1%	n.o.	13	44	0^c^	0^c^	0^c^	0
**Centre 7**	208	119	28	64%	n.o.	n.o.	4%	32%	1%	11	48	1	0	1	4^d^
**Centre 8**	1,300	206	41	50%	12%	23%	8%	6%	2%	12	41	1	0	1	10^b,e^
**Centre 9**	606 ^n.o.^	490	175	73%	0%	6%	8%	10%	3%	10	144	4	1.6	0	2^c^

n.o. = Modality is not offered at the unit. In some units, transplantation is not offered but carried out elsewhere. RRT = renal replacement therapy; HD = haemodialysis; HHD = home HD; PD = peritoneal dialysis; APD = automated PD; CAPD = continuous ambulatory PD; Asst. PD = assisted PD.

^a^ Pharmacists.

^b^ Technicians.

^c^ Roles fulfilled by a local non-profit patient organisation.

^d^ Physiotherapists and occupational therapists.

^e^ Nursing assistants.

#### Patient population

In 2012, there was a large range in the numbers of prevalent (90–793) and incident (25–175) dialysis patients between the different renal units. Full details are given in Table 1. The vast majority of incident patients received RRTOE (median 95%, range 60–100%). Of these patients, 97.5% completed the programme (range: 70–100%; 8 units responded to this question).

#### Uptakes of RRT modalities and RRTOE

The majority of prevalent patients (median 56%; range 50–94%) had received a transplant.

Of the dialysis patients, at least half were on in-centre HD at all renal units (range 50–95%). In centres offering home-based modalities, there were large differences in the percentages of patients receiving PD (median 20%; range 5–47%) or home HD (median 6%; range 0–23%). Full details are given in Table 1.

#### Staff in RRT unit

Numbers of staff at each unit are presented in Table 1. Almost all staff were nephrologists or nurses. Other staff included dieticians, psychologists, social workers, technicians, physiotherapists, occupational therapists and pharmacists.

### Section 2: Description of the RRTOE programme

#### Staff involved in RRTOE

Nurses were responsible for organising and providing RRTOE in 8 of the 9 units. However, other staff members were also involved in the RRTOE programme, such as nephrologists (7 units), dieticians (5 units), psychologists (4 units), social workers (3 units), physiotherapists (2 units), occupational therapists (1 unit), and pharmacists (1 unit). The percentage of their working time dedicated to RRTOE varied from centre to centre. Nephrologists spent 7.5% (median; range: 5–35%; 8 centres responded) of their working time on RRTOE. There were greater differences between units in the time spent on education by CKD nurses (median 45%; range: 5–100%; 8 centres) and PD nurses (median 5%; range 5–45%; 8 centres), with some units clearly making RRTOE the primary or sole function of some of their nurses.

All staff administering the programme had a background in general or nephrology nursing. Two centres had nurses with additional qualifications in adult education. Another two centres had nurses trained in motivational interviewing.

#### Starting RRTOE and enrolment criteria

In all units, patients began RRTOE several months before the need for dialysis or based on the level of disease progression. RRTOE participants generally included: patients with CKD stage IV or V (9 units); patients requiring a change in RRT treatment (8 units); family members of the patients (9 units).

#### Scheduling visits

**Figure 1:**
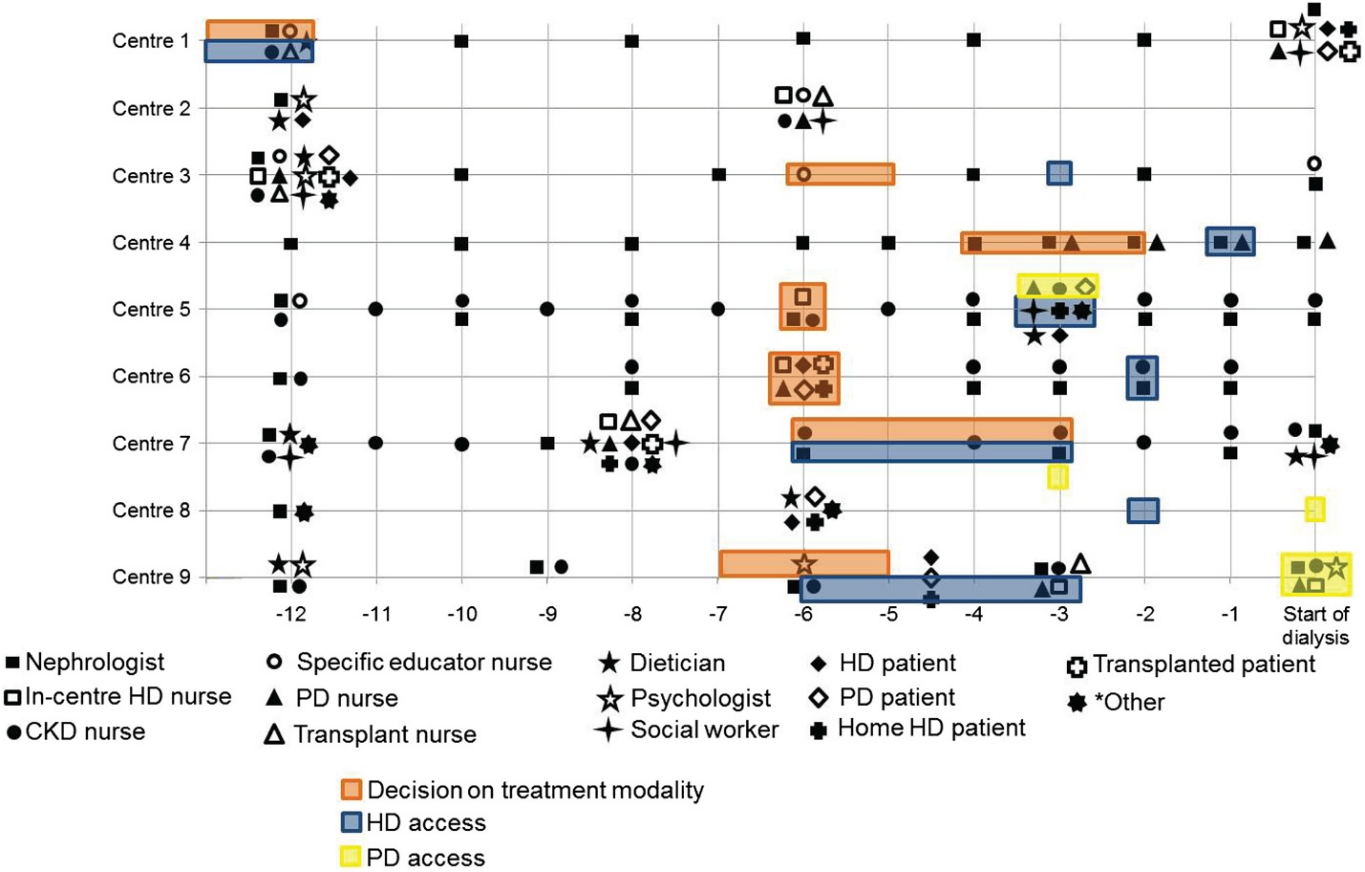
**Timing of meetings with HCPs and key decisions.** Orange, blue and yellow backgrounds indicate the times patients are expected to reach a decision on modality or have HD or PD access installed, respectively. Centre 3 also offers an optional 4-month course (sessions every 2 weeks) run by a multidisciplinary team for groups of 10 patients (with estimated glomerular filtration rates <20 ml/min per 1.73 m^2^) and their families. This is independent of expected treatment start. Centre 7 offers a residential course (2 nights) for 20 people (patients and families) twice per year. At Centre 1, patients have the option to meet with a transplant nurse, educational/pre-dialysis nurse, or dietician at each visit. Note that the figures for Centre 9 are approximations – patients are offered input based on an individual need basis. CKD = chronic kidney disease; HD = haemodialysis; PD = peritoneal dialysis.

The schedule of visits to HCPs at each centre is displayed in Figure 1. These visits are not specific to RRTOE. The majority of visits lasted between 15 and 60 minutes at all of the centres. Nephrologists and nurses are most often visited, usually on a 1- to 3-month basis.

#### Content and structure

**Figure 2:**
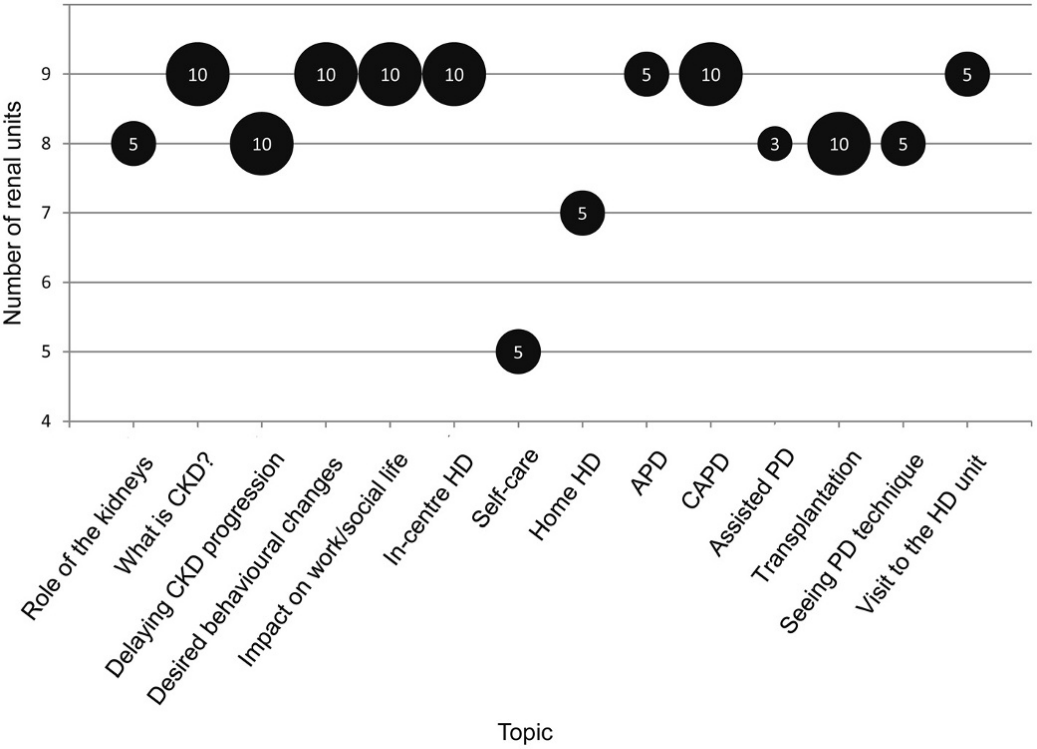
**Content of RRTOE.** X-axis = Topics covered in RRTOE. Y-axis = Number of renal units including this topic in their programme. Size of bubbles and numbers within = Median percentage of time (%) spent on this topic in centres including it in their RRTOE. CKD = chronic kidney disease; HD = haemodialysis; PD = peritoneal dialysis; APD = automated PD; CAPD = continuous ambulatory PD.

**Topics covered** The topics covered in the RRTOE programmes are presented in Figure 2. All or most units devoted around 10% of their programme time to topics such as CKD and desired behavioural changes. There was greater variation in the numbers of units teaching some or all of the dialysis modality options.

**Visits and meetings** In 8 of the 9 units, most RRT patients visit the in-centre HD unit. In 7 of the 9 units, most patients meet a home dialysis nurse to assess suitability for home treatment.

Formal meetings with an ‘expert patient’ are arranged in about half of the units as part of the RRTOE programme. One unit offers an interactive DVD featuring dialysis patients.

Education for groups of patients was undertaken in 3 of the 9 units.

**Formal decision-making process** 7 out of 9 centres have a formal decision-making process with written support materials in place. This process is generally administered by both nurses and nephrologists.

There was a wide range between centres in the typical time before starting dialysis that a patient would reach a decision on which modality to use (3–12 months). Full details are provided in Figure 1.

**Figure 3:**
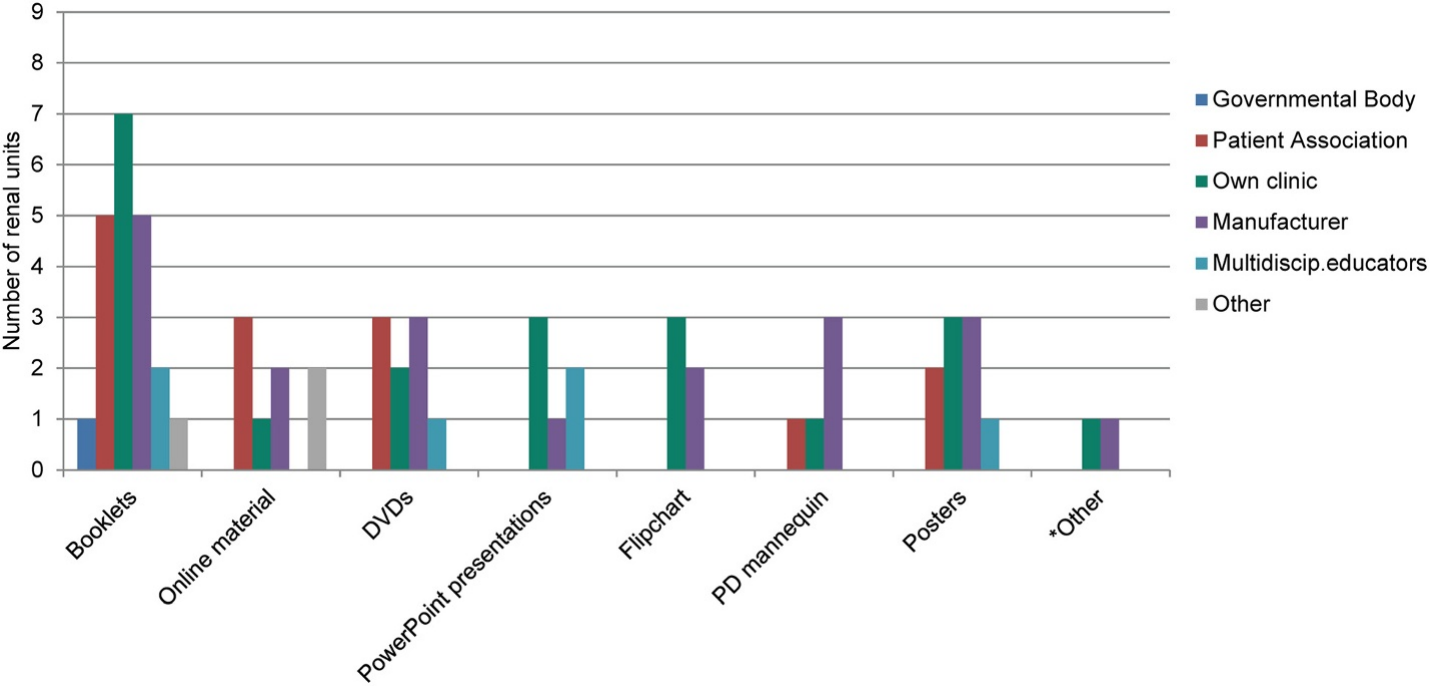
**Materials used in RRTOE and their source.** *Other: Option grids; agenda-setting cards; card game to clarify preferences; National Health Service’s decision aids online (UK only). PD, peritoneal dialysis.

**Materials** Booklets were used in all units as materials in the RRTOE. These came from a variety of sources (Figure 3). Online materials and DVDs were utilised by over half of the units.

**Setting** About half of the centres have a dedicated RRTOE room with visual aids.

#### Quality assurance

The most widely utilised measures of quality assurance (each used in 6 units) were: (1) patient satisfaction measured and used to improve the programme; (2) number of patients completing the programme; (3) linking the attendance/completion of the programme to clinical follow-up of the patient; (4) regularly updating materials.

Five units considered the number of patients on home-based therapy to be an indicator of RRTOE quality. Four units used the more formal educational approach of goal setting and progress evaluation.

#### Factors perceived to be important

The most widely agreed upon factors perceived as influencing the establishment and quality of RRTOE were national/local guidelines mandating RRTOE and the clinical leadership in the renal unit (each 6 units).

#### Funding

Eight of the 9 centres had no specific funding for their RRTOE programme. One unit had a dedicated budget within the hospital/unit.

## Discussion

### Results of the questionnaire

Considering that current guidelines on RRTOE are relatively broad, we expected to find large differences between the experts’ renal units in their approach to RRTOE. This was indeed the case.

Whilst nurses took responsibility for RRTOE in almost all units, there were striking differences in the utilisation of support staff such as dieticians, psychologists, social workers, and physical therapists. Some topics on RRTOE were presented in the programmes of all units, but only 4 units provided information on all treatment options.

Differences between the units were seen in the scheduling of visits to HCPs. However, the initial visit with a nephrologist was consistently around 12 months before initiation of dialysis. This is in line with US recommendations [14] and findings from a survey of Canadian nephrologists [19]. It should be noted that a retrospective chart review of 339 Canadian RRT patients [20] indicated that early referral is not necessarily associated with optimal dialysis start (defined as dialysis initiated as an outpatients with an arteriovenous fistula, arteriovenous graft or peritoneal dialysis catheter).

Materials used for RRTOE also differed between units, but perhaps the most concerning finding was the lack of agreement on how to objectively measure the quality of the RRTOE programme. This may reflect the many endpoints used in the literature, e.g. number of patients with permanent vascular access [7], reduced time spent in hospital [6] and initiation of home-based dialysis [8].

The lack of standardisation found here – even between renal units with specialists in this area – indicates that the consensus statement [17] produced from this meeting will provide helpful guidance to RRTOE teams. However, strong evidence of the need for guidelines comes from a recent literature review of predialysis education programmes (Van den Bosch et al., Review of Predialysis Education Programmes: a Need for Standardization. Submitted). This review concluded that the lack of standardisation between such programmes makes it very difficult to evaluate their effectiveness on a large scale.

It should be stressed that additional forms of patient support to the ones already mentioned can be offered. For example, RRTOE could incorporate factors to increase health-related quality of life, such as dealing with psychological problems arising from the mental suffering, reduced vitality, and lack of socialisation often experienced during the course of the disease [21] or from changes in treatment [22, 23]. Coping strategies for combating non-adherence to medication would also be beneficial [24]. Moreover, there is evidence that modality choice can impact the emotional status and coping of caregivers [25]. As caregivers are encouraged to attend RRTOE, it may be beneficial to have some programme content tailored towards their needs.

There are some limitations to our targeted survey. The number of participating renal units was low. Also, as the centres were chosen because they had extensive experience with RRTOE or employed a researcher in this area, they were not representative examples of RRTOE in Europe. Thus, the data presented here cannot be used to infer the effectiveness of RRTOE.

### Feedback on the questionnaire

The ultimate purpose of this questionnaire is to help fill the knowledge gap on how RRTOE is being carried out across Europe. During and after the meeting, the experts made substantial modifications to the questionnaire to make it suitable for large-scale roll-out.

The main change is that the format has been altered to allow comparison with the guidance in the consensus statement [17]. This will allow both participants and researchers to quickly compare each unit’s results to current best practice. Other changes include: (1) shortening the time required for completion; (2) accounting for substantial differences in roles/responsibilities between staff with identical job titles; (3) giving clear definitions to avoid confusion between RRTOE and education that follows modality choice (e.g. giving instructions on changing a bag in PD).

This survey is expected to commence mid-2014. It is anticipated that the resulting data will be of interest to all HCPs in this area, as well as researchers and policy-makers.

## Conclusions

Results from our pilot questionnaire highlight the wide variety of approaches taken to educating patients on their renal replacement options. Even renal units in close geographical proximity use different criteria to initiate RRTOE, use different types of HCPs to conduct the training, provide different materials, and use different quality measures. Therefore, the consensus statement produced from this meeting will be of value to all renal teams running such RRTOE programmes. The upcoming field survey will provide detailed information on how closely RRTOE procedures adopted by renal units across Europe match the recommendations in the consensus statement.

## Electronic supplementary material

Additional file 1: **Questionnaire for renal units.** (PDF 429 KB)

## Abbreviations

APD: Automated peritoneal dialysis
CAPD: Continuous ambulatory peritoneal dialysis
CKD: Chronic kidney disease
ESRD: End-stage renal disease
HCPs: Health care practitioners
HD: Haemodialysis
PD: Peritoneal dialysis
RRT: Renal replacement therapy
RRTOE: Renal replacement therapy option education.
