# Supplementary material for: Predialysis education in practice: a questionnaire survey of centres with established programmes
Source: BMC Res Notes. 2014 Oct 17;7:730. doi: 10.1186/1756-0500-7-730 (PMC4210595; doi:10.1186/1756-0500-7-730)
Supplement: Supplementary file 1 — Additional file 1: Questionnaire for renal units. (PDF 429 KB) [file 13104_2014_3255_MOESM1_ESM.pdf]

EXPERT SURVEY

INTRODUCTION

Dear Expert Committee member,

Thank you for agreeing to attend the meeting on Predialysis/Renal Replacement Therapy (RRT) Option Education. To help our discussions on the day, we would be grateful if you could complete this questionnaire about your own programme, as fully as you can. We ask you to kindly complete this by 15 March 2013. We can then look at the summarised results at the meeting.

The questionnaire is divided into 2 sections.
- The first section is a description of your renal unit, its services and its patient population in end stage renal disease (ESRD).
- The second section covers your Predialysis/RRT Option Education programme.

We thank you in advance for your help.

***What is your name?**

SECTION 1 - YOUR RENAL UNIT, ITS SERVICES AND ITS PATIENT POPULATION IN END...

A- The dialysis services

***a. The following modalities are delivered at my hospital:**

- ☐ In-centre haemodialysis (HD), including satellite unit dialysis
- ☐ Self-care HD
- ☐ Home HD
- ☐ Automated peritoneal dialysis (APD)
- ☐ Continuous ambulatory peritoneal dialysis (CAPD)
- ☐ Assisted PD
- ☐ Transplantation (surgical procedure)

SECTION 1 - YOUR RENAL UNIT, ITS SERVICES AND ITS PATIENT POPULATION IN END...

B- The patients

*a. Please indicate the number of prevalent renal replacement therapy (RRT) patients on 31 December 2012.

Total

In-centre HD, including satellite unit dialysis

Self-care HD

Home HD

APD

CAPD

Assisted PD

Transplant

*b. Please indicate the number of incident dialysis patients in 2012.

Total

In-centre HD, including satellite unit dialysis

Self-care HD

Home HD

APD

CAPD

Assisted PD

*c. Of your new dialysis patients in 2012, how many do you think received Predialysis/RRT Option Education? (%)

Total

In-centre HD, including satellite unit dialysis

Self-care HD

Home HD

APD

CAPD

Assisted PD

***d. How many of those patients do you think completed the programme? (%)**

Total	
In-centre HD, including satellite unit dialysis	
Self-care HD	
Home HD	
APD	
CAPD	
Assisted PD	

SECTION 1 - YOUR RENAL UNIT, ITS SERVICES AND ITS PATIENT POPULATION IN END...

C- Your Renal Unit

Please give some details of your renal unit. How many full time equivalents of each of the healthcare professionals listed here does your unit have?
Please tell us the percentage of their total time allocated to the renal unit, to one or more dialysis modalities, and if they rotate between roles.

*a. How many nephrologists (full time equivalents) does your unit have? Please tell us the percentage of their total time allocated to the renal unit, to one or more dialysis modalities, and if they rotate between roles.

Total (n)	
CKD clinic / predialysis care (%)	
In-centre HD, including satellite unit dialysis (%)	
Self-care HD (%)	
HHD (%)	
PD (%)	
Predialysis/ RRT Option Education (%)	
Rotation between roles (Please enter "1" for yes and "0" for no)	

*b. How many nurses (full time equivalents) does your unit have? Please tell us the percentage of their total time allocated to the renal unit, to one or more dialysis modalities, and if they rotate between roles.

Total (n)	
CKD clinic / predialysis care (%)	
In-centre HD, including satellite unit dialysis (%)	
Self-care HD (%)	
HHD (%)	
PD (%)	
Predialysis/ RRT Option Education (%)	
Rotation between roles (Please enter "1" for yes and "0" for no)	

***c. How many dieticians (full time equivalents) does your unit have? Please tell us the percentage of their total time allocated to the renal unit, to one or more dialysis modalities, and if they rotate between roles.**

Total (n)

CKD clinic / predialysis care (%)

In-centre HD, including satellite unit dialysis (%)

Self-care HD (%)

HHD (%)

PD (%)

Predialysis/ RRT Option Education (%)

Rotation between roles (Please enter "1" for yes and "0" for no)

***d. How many psychologists (full time equivalents) does your unit have? Please tell us the percentage of their total time allocated to the renal unit, to one or more dialysis modalities, and if they rotate between roles.**

Total (n)

CKD clinic / predialysis care (%)

In-centre HD, including satellite unit dialysis (%)

Self-care HD (%)

HHD (%)

PD (%)

Predialysis/ RRT Option Education (%)

Rotation between roles (Please enter "1" for yes and "0" for no)

***e. How many social workers (full time equivalents) does your unit have? Please tell us the percentage of their total time allocated to the renal unit, to one or more dialysis modalities, and if they rotate between roles.**

Total (n)

CKD clinic / predialysis care (%)

In-centre HD, including satellite unit dialysis (%)

Self-care HD (%)

HHD (%)

PD (%)

Predialysis/ RRT Option Education (%)

Rotation between roles (Please enter "1" for yes and "0" for no)

***f. How many other healthcare professionals (full time equivalents) does your unit have?**
Please tell us the percentage of their total time allocated to the renal unit, to one or more dialysis modalities, and if they rotate between roles.

Total (n)

CKD clinic / predialysis care (%)

In-centre HD, including satellite unit dialysis (%)

Self-care HD (%)

HHD (%)

PD (%)

Predialysis/ RRT Option Education (%)

Rotation between roles (Please enter "1" for yes and "0" for no)

g. If your unit includes other healthcare professionals, could you please specify?

h. Is there anything else you would like to tell us about your renal unit?

SECTION 2 - DESCRIPTION OF THE PREDIALYSIS/RRT OPTION EDUCATION PROGRAMME

D- The process

At our hospital:

***a. Patient education on RRT options typically starts (tick all those that apply):**

- ☐ Based on the level of disease progression
- ☐ Several months before the need for dialysis
- ☐ As the patient is referred to us for dialysis

SECTION 2 - DESCRIPTION OF THE PREDIALYSIS/RRT OPTION EDUCATION PROGRAMME

***You chose the option "Based on the level of disease progression". Could you please specify the trigger point?**

SECTION 2 - DESCRIPTION OF THE PREDIALYSIS/RRT OPTION EDUCATION PROGRAMME

*b. Education on RRT options is organised and provided by (tick all that apply):

- ☐ In-centre (hospital) HD nurse
- ☐ CKD nurse
- ☐ PD nurse
- ☐ A specific educator nurse
- ☐ Other (please specify)

*c. For typical patients who are referred well ahead of the start of dialysis treatment, how many months before the start of dialysis do visits with the following professionals / patient experts take place?

	Month - 12	Month - 11	Month - 10	Month - 9	Month - 8	Month - 7	Month - 6	Month - 5	Month - 4	Month - 3	Month - 2	Month - 1	Start of dialysis
Nephrologist	☐	☐	☐	☐	☐	☐	☐	☐	☐	☐	☐	☐	☐
In-centre (hospital) HD nurse	☐	☐	☐	☐	☐	☐	☐	☐	☐	☐	☐	☐	☐
CKD nurse	☐	☐	☐	☐	☐	☐	☐	☐	☐	☐	☐	☐	☐
Specific educator nurse	☐	☐	☐	☐	☐	☐	☐	☐	☐	☐	☐	☐	☐
PD nurse	☐	☐	☐	☐	☐	☐	☐	☐	☐	☐	☐	☐	☐
Transplant nurse	☐	☐	☐	☐	☐	☐	☐	☐	☐	☐	☐	☐	☐
Dietician	☐	☐	☐	☐	☐	☐	☐	☐	☐	☐	☐	☐	☐
Psychologist	☐	☐	☐	☐	☐	☐	☐	☐	☐	☐	☐	☐	☐
Social worker	☐	☐	☐	☐	☐	☐	☐	☐	☐	☐	☐	☐	☐
HD patient	☐	☐	☐	☐	☐	☐	☐	☐	☐	☐	☐	☐	☐
PD patient	☐	☐	☐	☐	☐	☐	☐	☐	☐	☐	☐	☐	☐
Home HD patient	☐	☐	☐	☐	☐	☐	☐	☐	☐	☐	☐	☐	☐
Transplanted patient	☐	☐	☐	☐	☐	☐	☐	☐	☐	☐	☐	☐	☐
*Other	☐	☐	☐	☐	☐	☐	☐	☐	☐	☐	☐	☐	☐

*Please specify other

d. What is the average duration of a visit (expressed in minutes)?

Nephrologist	
In-centre (hospital) HD nurse	
CKD nurse	
Specific educator nurse	
PD nurse	
Transplant nurse	
Dietician	
Psychologist	
Social worker	
HD patient	
PD patient	
Home HD patient	
Transplanted patient	
*Other	

SECTION 2 - DESCRIPTION OF THE PREDIALYSIS/RRT OPTION EDUCATION PROGRAMME

E- The content

***a. The Predialysis/RRT Option Education Programme in my unit covers the following aspects (please provide an estimate of what percentage of the education programme is dedicated to each of the elements; e.g., for 10% enter 10; type 0 if the element is not covered; n.b.: the total must equate to 100):**

The role of the kidneys	
What is CKD and what does it mean for the patient in the future (i.e., impact on his/her life and activities)	
Means to delay the progression of the disease	
Necessary/desired behavioural changes (e.g., diet, physical exercises)	
Impact on ability to work and/or income, family and social life	
In-centre HD	
Self-care HD	
Home HD	
APD	
CAPD	
Assisted PD	
Transplantation	
Seeing the PD technique	
Visits to HD unit	

SECTION 2 - DESCRIPTION OF THE PREDIALYSIS/RRT OPTION EDUCATION PROGRAMME

E- The content

*b1. Do most of your planned start patients :

	Yes	No
See a home dialysis nurse (PD or Home HD) to assess suitability for home treatment	○	○
See a social worker to assess suitability for home treatment	○	○
See a psychologist to assess suitability for home treatment	○	○
Visit the in-centre HD unit	○	○
Visit the self-care HD unit	○	○

*b2. Do most of your planned start patients have a formal meeting arranged with other patients:

	Yes	No
In-centre HD patient	○	○
Self-care HD patient	○	○
Home HD patient	○	○
PD patient	○	○

b3. If there are any other visits or meetings scheduled for planned start patients, could you please specify?

SECTION 2 - DESCRIPTION OF THE PREDIALYSIS/RRT OPTION EDUCATION PROGRAMME

E- The content

***c. Do you have a formal patient decision making process in place with written decision support tools?**

- Yes
- No

SECTION 2 - DESCRIPTION OF THE PREDIALYSIS/RRT OPTION EDUCATION PROGRAMME

E- The content

***d. If yes, please describe (e.g. Which team members are involved? How do you proceed? etc.):**

SECTION 2 - DESCRIPTION OF THE PREDIALYSIS/RRT OPTION EDUCATION PROGRAMME

F- The format

***a. Please tell us which materials you use for the Predialysis/RRT Option Education Programme (tick all that apply) and where the materials were developed or provided from:**

	Tick if used	Governmental body	Patient association	Own clinic	Manufacturer (pharma or medical device)	Multidisciplinary educators	*Other	Tick if given to the patient
Booklets	☐	☐	☐	☐	☐	☐	☐	☐
Online material	☐	☐	☐	☐	☐	☐	☐	☐
DVDs	☐	☐	☐	☐	☐	☐	☐	☐
PowerPoint presentations	☐	☐	☐	☐	☐	☐	☐	☐
Flipchart	☐	☐	☐	☐	☐	☐	☐	☐
PD mannequin	☐	☐	☐	☐	☐	☐	☐	☐
Posters	☐	☐	☐	☐	☐	☐	☐	☐
*Other	☐	☐	☐	☐	☐	☐	☐	☐

*Please specify any other material (Booklet, Online Material, etc.) and/or other source of material (Governmental body, Patient association, etc.) used.

SECTION 2 - DESCRIPTION OF THE PREDIALYSIS/RRT OPTION EDUCATION PROGRAMME

G- The Settings

a. The Predialysis/RRT Option Education Programme is delivered in the following settings (tick all that apply – please send a picture to Elly Gysels at archimed if available)

- ☐ Dedicated room with visual aids (e.g., printed material) on dialysis options
- ☐ Doctor's clinical room
- ☐ Any room that is available
- ☐ Other (please specify)

SECTION 2 - DESCRIPTION OF THE PREDIALYSIS/RRT OPTION EDUCATION PROGRAMME

H- The patients

***a. Patients who take part in the Predialysis/RRT Option Education Programme include (tick all that apply)**

- [ ] CKD stage 3-4 patients
- [ ] Unplanned start patients
- [ ] ESRD patients requiring a change in their RRT treatment
- [ ] The family members of the patients
- [ ] Other (please specify)

***b. Do you have a formal programme to discuss dialysis options with existing dialysis patients?**

- ○ Yes
- ○ No

SECTION 2 - DESCRIPTION OF THE PREDIALYSIS/RRT OPTION EDUCATION PROGRAMME

H- The patients

***c. If yes, is it the same as for predialysis patients?**

- Yes
- No

SECTION 2 - DESCRIPTION OF THE PREDIALYSIS/RRT OPTION EDUCATION PROGRAMME

H- The patients

***d. If no, please describe the differences with your predialysis programme:**

SECTION 2 - DESCRIPTION OF THE PREDIALYSIS/RRT OPTION EDUCATION PROGRAMME

I- The funding

*a. The funding arrangements of the Predialysis/RRT Option Education Programme include (tick all that apply)

- [ ] The programme has a dedicated budget within the hospital/unit
- [ ] There is an official external funding process (e.g., through the reimbursement system) to the hospital/clinic for the Predialysis/RRT Option Education Programme
- [ ] The Predialysis/RRT Option Education Programme is funded through charity
- [ ] There is no specific funding for the Predialysis/RRT Option Education Programme
- [ ] Other (please specify)

SECTION 2 - DESCRIPTION OF THE PREDIALYSIS/RRT OPTION EDUCATION PROGRAMME

J- The staff

***a. Who is involved in the Predialysis/RRT Option Education Programme? Please indicate the number of people.**

Nephrologist

In-centre HD nurse

CKD nurse

PD nurse

A specific education nurse

Dietician

Psychologist

Social worker

*Other

*** If you selected "other", please specify:**

b. What is the percentage of their time allocated to Predialysis/RRT Option Education?

Nephrologist (%)

In-centre HD nurse (%)

CKD nurse (%)

PD nurse (%)

A specific education nurse (%)

Dietician (%)

Psychologist (%)

Social worker (%)

*Other

c. If the persons involved in the Predialysis/RRT Option Education Programme have other roles, please specify them here.

Nephrologist	
In-centre HD nurse	
CKD nurse	
PD nurse	
A specific education nurse	
Dietician	
Psychologist	
Social worker	
*Other	

SECTION 2 - DESCRIPTION OF THE PREDIALYSIS/RRT OPTION EDUCATION PROGRAMME

K- Nursing professionals' skills

This is about the nurses who are involved in Predialysis/RRT Option Education of your Unit. Please describe their professional skills.

*a. Educational background:

- [ ] General nursing
- [ ] Nephrology nursing
- [ ] Other (please specify)

*b. Main role:

- [ ] PD nurse
- [ ] Home HD nurse
- [ ] Predialysis/RRT Option Education
- [ ] CKD nurse
- [ ] In-centre HD nurse
- [ ] Self-care HD nurse
- [ ] Other (please specify)

*c. Expertise in adult learning obtained (tick all that apply)

- [ ] Official qualification
- [ ] Through practice
- [ ] None
- [ ] Other (please specify)

SECTION 2 - DESCRIPTION OF THE PREDIALYSIS/RRT OPTION EDUCATION PROGRAMME

K- Nursing professionals’ skills

***d. You selected "Official qualification" in the previous question. Please specify:**

Name of qualification:

From which institution:

SECTION 2 - DESCRIPTION OF THE PREDIALYSIS/RRT OPTION EDUCATION PROGRAMME

L- Quality indicators

*a. Check the following quality indicators about Predialysis/RRT Option Education that are routinely measured at your hospital

- [ ] Learning goals are set for each patient and achievement of the goals is assessed at each encounter
- [ ] Patient education material is updated on a regular basis (i.e., at least every 2 years)
- [ ] Patient satisfaction with the programme is measured and used to improve the programme
- [ ] Number of patients on home-based therapies above a defined level (e.g. 10%)
- [ ] Number of patients completing programme vs. defined standard (e.g. 75% of all planned start patients should complete course programme)
- [ ] The RRT programme attendance and completion is linked to the clinical follow-up of the CKD patient through e.g. an electronic health record or registration on a database
- [ ] Other (please specify)

SECTION 2 - DESCRIPTION OF THE PREDIALYSIS/RRT OPTION EDUCATION PROGRAMME

M- Elements influencing the success of your programme

***a. In your opinion, which elements influence the success of your Predialysis/RRT Option Education programme (tick all that apply):**

- ☐ National/regional guidelines mandating treatment option education
- ☐ Reimbursement of treatment option education
- ☐ Active patient organisation(s) lobbying for unbiased patient choice
- ☐ Clinical leadership in CKD clinic and or dialysis unit
- ☐ Other (please specify)

***b. Since the introduction of your Predialysis/RRT Option Education programme, what significant changes have you made and why? (e.g. different staff, new material)**

***c. Is there anything else you might want to tell us that you feel is important to the success of your home programme?**
